# Successful Postnatal Management of Ruptured Giant Sacrococcygeal Teratoma

**DOI:** 10.21699/jns.v6i2.471

**Published:** 2017-04-15

**Authors:** Muataz A. Alani

**Affiliations:** Department of Pediatric Surgery, Central Pediatric Teaching Hospital, Baghdad, Iraq.

**Keywords:** Sacrococcygeal teratoma, In-utero, Rupture

## Abstract

Most sacrococcygeal teratomas present between the 22nd and the 34th week of gestation. The diagnosis of sacrococcygeal teratoma on routine antenatal sonograms is associated with a greater than expected incidence of prenatal and perinatal complications. We report a premature baby with intrauterine spontaneous rupture of giant sacrococcygeal teratoma which was managed successfully.

## Case Report

**Figure 1:**
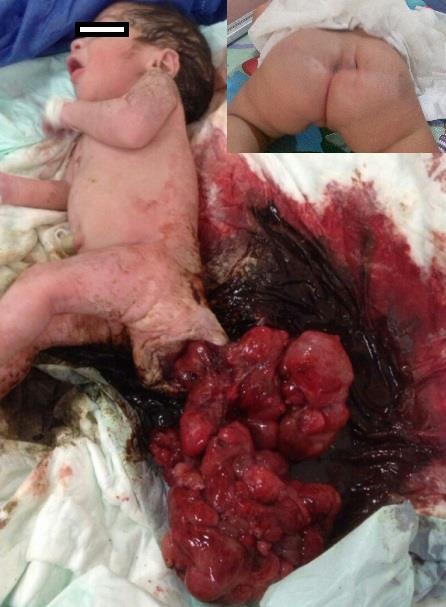
Showing ruptured SCT. Inset showed healed scar at 6-month follow-up.

A 28-year-old, G3P2 mother delivered a female at 35-week of gestational age, without proper antenatal sonographic follow up, the newborn baby weighed 3200 gm (including the weight of the tumor) via emergency cesarean section (CS) due to fetal distress. At CS more than usual bloody amniotic fluid was noticed. The newborn had an APGAR scores of 3 and 7 at 1 and 5 min, respectively. The infant was very pale and had 18×11×16cm, well-vascularized lacerated mass at the coccygeal region (Fig.1). After a period of resuscitation, surgical resection of the tumor was carried out on her first neonatal day. There was evidence of minor bleeding from the mass with evidence of necrosis within the tumor. The tumor weighed 1050 gm. Pathological examination showed an immature teratoma with malignant foci. Postoperatively, the baby had uneventful recovery. Patient was referred to oncologist for chemotherapy. The patient is under our follow-up and doing fine with normal range of alpha-fetoprotein.

## Discussion

Fetuses with huge sacrococcygeal teratoma may undergo preterm delivery due to uterine over distention or associated polyhydramnios and have a high risk of perinatal complications and death [1-3]. Neonatal death may result from maternal obstetric complications of tumor rupture, preterm labor, or dystocia [1]. Tumor rupture may be caused by uncontrolled labor or complications during delivery [1] Hemorrhagic mortality of neonates with SCT is relatively high (3.8%). High-output cardiac failure, internal tumor hemorrhage and perioperative bleeding are the most common causes of early death and were all strongly associated with larger tumor sizes [4,5]. We succeeded in the management of our patient as the rupture was associated with insignificant bleeding. During surgery, precautions must be taken to prevent hypothermia, which is easily precipitated because of the large surface area and the vascularity of the tumor.

## Footnotes

**Source of Support:** None

**Conflict of Interest:** None
